# Anti-cholinesterases and memory improving effects of Vietnamese *Xylia xylocarpa*

**DOI:** 10.1186/s13065-016-0197-5

**Published:** 2016-08-03

**Authors:** Linh My Thi Lam, Mai Thanh Thi Nguyen, Hai Xuan Nguyen, Phu Hoang Dang, Nhan Trung Nguyen, Hung Manh Tran, Hoa Thi Nguyen, Nui Minh Nguyen, Byung Sun Min, Jeong Ah Kim, Jae Sue Choi, Mao Van Can

**Affiliations:** 1 Faculty of Chemistry, University of Science, Vietnam National University-Hochiminh City, 227 Nguyen Van Cu, District 5, Hochiminh City, Vietnam; 2 Vietnam Military Medical University, Hadong District, Hanoi, Vietnam; 3 College of Pharmacy, Catholic University of Daegu, Gyeongsan, Gyeongsangbuk 712-702 Republic of Korea; 4 College of Pharmacy, Research Institute of Pharmaceutical Sciences, Kyungpook National University, Daegu, 702-701 Republic of Korea; 5 Department of Food Science and Nutrition, Pukyong National University, Busan, 608-737 Republic of Korea; 6 Cancer Research Laboratory, Vietnam National University-Hochiminh City, Hochiminh City, Vietnam

**Keywords:** *Xylia xylocarpa*, Hopan-ol-olide, Acetylcholinesterase, Butyrylcholinesterase, Improving memory effects

## Abstract

**Background:**

Alzheimer’s disease (AD) is the most common cause of dementia among the elderly and is characterized by loss of memory and other cognitive functions. An increase in AChE (a key enzyme in the cholinergic nervous system) levels around *β*-amyloid plaques and neurofibrillary tangles is a common feature of AD neuropathology. Amnesic effects of scopolamine (acetylcholine receptor antagonist) can be investigated in various behavioral tests such as Morris water maze, object recognition, Y-maze, and passive avoidance. In the scope of this paper, we report the anti-AChE, anti-BChE properties of the isolated compound and the in vivo effects of the methanolic extract of *Xylia xylocarpa* (MEXX) on scopolamine-induced memory deficit.

**Results:**

In further phytochemistry study, a new hopan-type triterpenoid, (3*β*)-hopan-3-ol-28,22-olide (**1**), together with twenty known compounds were isolated (**2**–**21**). Compound **1**, **2**, **4**, **5**, **7**–**9**, and **11**–**13** exhibited potent acetylcholinesterase (AChE) inhibitory activity in a concentration-dependent manner with IC_50_ values ranging from 54.4 to 94.6 μM. Compound **13** was also shown anti-butyrylcholinesterase (BChE) activity with an IC_50_ value of 42.7 μM. The Morris water Y-maze, Y-maze, and object recognition test were also carried out.

**Conclusions:**

It is noteworthy that MEXX is effective when administered orally to mice, experimental results are consistent with the traditional use of this medicinal plant species.

**Electronic supplementary material:**

The online version of this article (doi:10.1186/s13065-016-0197-5) contains supplementary material, which is available to authorized users.

## Background

Alzheimer’s disease (AD), a degenerative brain disorder leading to dementia, is one of the most common disorders of old age, affecting nearly 4 million individuals in the US. Typical clinical features of Alzheimer’s disease are memory loss, language deterioration, reduced visual space, sensation disorders and epilepsy advocacy gradual progression of terminal illness [1, 2]. There are several theories about the cause of Alzheimer’s disease, in which the theory about the decline of acetylcholine is the most widely accepted and is the basis for the current development of the drugs of Alzheimer’s disease. The research on Alzheimer’s patients demonstrated that cholinergic abnormalities correlated with the degree of memory and cognitive impairment [2, 3]. These findings have led to the treatment of Alzheimer’s disease by increasing the activity of the cholinergic system (acetylcholinesterase, AChE, inhibitory mechanism) [2, 3]. Recently, some research found that AChE is also related to the formation of amyloid plaques and neurofibrillary tangles [4].

*Xylia xylocarpa* (Roxb.) Taub. is a perennial tree belonging to the family Fabaceae, which is sparsely distributed in Burma, Vietnam, Cambodia, and India. In Vietnam, *X. xylocarpa* is known as “Cam Xe”; the bark, heartwood, and flower have been used as Vietnamese traditional medicines for the treatment of dementia, duodenal, stomach pain, vomiting, diarrhoea, gonorrhoea, leprosy, and rheumatism [5]. Previously, the chemical constituents of the wood of *X. xylocarpa* have been reported some flavan-3-ols including monomer, dimer, and trimer of epiafzelechin [6]. Our preliminary screening study also revealed that the methanolic extract of the wood of *X. xylocarpa* exhibited significant AChE and BChE (butyrylcholinesterase) inhibitory activities with IC_50_ values of 16.17 and 7.13 μg/mL, respectively. In the present study, we report the cognitive-enhancing effect of the methanolic extract of *X. xylocarpa* (MEXX) on amnesic mice induced by scopolamine in vivo. In addition, the isolation of MEXX was carried out, a new hopan-type triterpenoid, (3*β*)-hopan-3-ol-28,22-olide (**1**) was isolated together with twenty known compounds (**2**–**21**). We also reported the anti-AchE, anti-BChE properties of the isolated compound herein.

## Results and discussions

### Chemistry

**Fig. 1:**
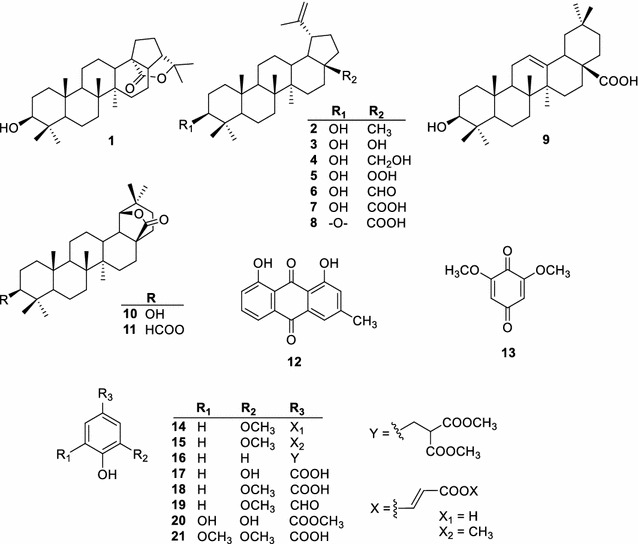
Chemical structures of isolated compounds (**1**–**21**) from the wood of *X. xylocarpa*

The MEXX was suspended in H_2_O and then successively partitioned with hexane, EtOAc, and BuOH to yield hexane, EtOAc, BuOH and H_2_O fractions, respectively. Separation and purification of EtOAc soluble fraction led to the isolation of a new hopan-ol-olide named (3*β*)-hopan-3-ol-28,22-olide (**1**), together with twenty known compounds (**2**–**21**). These known compounds were identified as lupeol (**2**) [7]; 28-norlup-20(29)-ene-3*β*,17*β*-diol (**3**) [8]; betulin (**4**) [9]; 28-norlup-20(29)-ene-3*β*-hydroxy-17β-hydroperoxide (**5**) [10]; betulinaldehyde (**6**) [11]; betulinic acid (**7**) [12]; betulonic acid (**8**) [12]; oleanolic acid (**9**) [13]; 3*β*-hydroxy-18*α*-olean-28,19*β*-olide (**10**) [14]; 3*β*-formyloxy-l8α-oleanano-28,19β-lactone (**11**) [15]; chrysophanol (**12**) [16]; 2,6-dimethoxyl-*p*-benzoquinone (**13**) [17]; ferulic acid (**14**) [18]; methyl ferulate (**15**) [19]; methyl 3-(4-hydroxyphenyl)-2-methoxycarbonylpropionate (**16**) [20]; protocatechuic acid (**17**) [21]; vanillic acid (**18**) [22]; vanillin (**19**) [23]; methyl gallate (**20**) [24]; and syringic acid (**21**) [22] (Fig. 1) based on the spectroscopic analysis and comparison with literature data.

Compound **1** exhibited an [M + H]^+^ and [M + Na]^+^ peak at *m/z* 457.3674 and 479.3482, respectively, in the positive HR-ESI-MS, corresponding to the molecular formula C_30_H_48_O_3_. The ^13^C NMR spectrum of compound **1** showed thirty carbon signals, including one lactone carbonyl carbon (*δ*_C_ 175.9), one hydroxylated methine (*δ*_C_ 79.1), and one oxygenated tertiary carbon (*δ*_C_ 83.4). Together with the HSQC analysis, all the remaining carbon signals were identified as five methines, ten methylenes, five quaternary carbons and seven tertiary methyl groups. The ^1^H NMR spectrum of compound **1** also exhibited an oxygenated methine proton signal at *δ*_H_ 3.19 (dd, *J* = 11.4 and 4.8 Hz, H-3) and seven singlet methyl signals (*δ*_H_ 1.46, 1.33, 0.96, 0.94, 0.93, 0.83, 0.76). Based on the analysis of these spectra, compound **1** was suggested to be an hopan-type triterpenoid [25, 26].

**Fig. 2:**
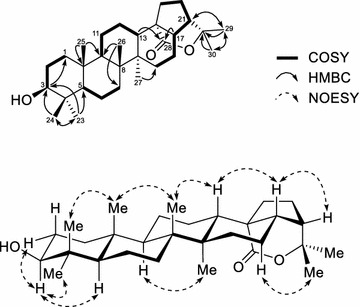
The selected ^1^H-^1^H COSY, HMBC and NOESY correlations of **1**

The location of hydroxyl group was deduced to be at C-3, based on the HMBC correlations between the oxygenated methine proton H-3 and the methylene carbon C-1 (*δ*_C_ 39.1). The HMBC cross-peaks from Me-23 (*δ*_H_ 0.96) and Me-24 (*δ*_H_ 0.76) to the hydroxylated carbon C-3 (*δ*_C_ 79.1); and the splitting patterns of proton H-3 also indicated the hydroxyl group was attached to C-3. The ester carbonyl group was located at C-28 due to the HMBC correlations between the methine proton H-13/H-17 and the carbonyl carbon C-28. The tertiary methyl protons H-29 and H-30 exhibited simultaneously HMBC correlations with the oxygenated tertiary carbon (*δ*_C_ 83.4), these was carbon C-22. Based on the chemical shift of C-22 and C-28 [25], it is clear that the lactone ring was formed between these carbons. Combining the ^1^H- and ^13^C NMR data (Table 1) with the HSQC, COSY and HMBC analysis (Fig. 2), the skeletal structure of **1** was confirmed as a hopan-3-ol-28,22-olide. The proton H-3 appeared as a doublet of doublets (*δ*_H_ 3.19, *J* = 11.4 and 4.8 Hz) that indicating an axial position of this proton. In the NOESY spectrum (Fig. 2), the correlated signals were observed between H-3/equatorial H-2, H-3/H-5, H-3/H-23 indicating that the 3-OH group was *β*-equatorial orientation. The NOESY spectrum also exhibited the correlations of H-24/H-25, H-25/H-26, H-13/H-26, and H-9/H-27; these observations confirmed four rings A, B, C, and D were *trans*-fused. The NOE correlations between H-13/H-17 and H-17/H-21 confirmed the *β*-equatorial orientation of H-21. Thus, the structure of compound **1** was elucidated to be (3*β*)-hopan-3-ol-28,22-olide.

**Table 1:** ^1^H and ^13^C NMR data for (3*β*)-hopan-3-ol-28,22-olide (**1**) in CDCl_3_

Position	(3*β*)-Hopan-3-ol-28,22-olide (**1**)	
	*δ*_C_, type	*δ*_H_ *(J* in Hz*)*
1a	39.1, CH_2_	1.62, m
1b		1.72, m
2	27.6, CH_2_	1.61, m
3	79.1, CH	3.19, dd (11.4, 4.8)
4	41.0, C	–
5	55.6, CH	0.69, m
6	18.5, CH_2_	1.56, m
7	34.2, CH_2_	1.39, m
8	41.8, C	–
9	50.9, CH	1.38, m
10	37.4, C	–
11	20.6, CH_2_	1.51, m
12	27.0, CH_2_	1.62, m
13	37.1, CH	1.79, m
14	41.8, C	–
15a	33.8, CH_2_	1.82, m
15b		1.59, m
16a	26.5, CH_2_	2.00, m
16b		1.61, m
17	48.3, CH	1.62, m
18	48.7, C	–
19a	29.1, CH_2_	2.41, dt (13.3, 3.5)
19b		1.25–1.30, m
20	29.1, CH_2_	1.25, m
21	42.6, CH	2.13, t (4.4)
22	83.4, C	–
23	28.2, CH_3_	0.96, s
24	15.9, CH_3_	0.76, s
25	16.4, CH_3_	0.83, s
26	15.5, CH_3_	0.93, s
27	14.2, CH_3_	0.94, s
28	175.9, C	–
29	30.3, CH_3_	1.46, s
30	30.4, CH_3_	1.32, s

### Biological assay

The isolated compounds were tested for their AChE and BChE inhibitory activities at various concentrations using berberin, a known inhibitor of AchE isolated from many plant species, as a positive control (Table 2). In the AChE inhibition assay, compounds **1**, **2**, **4**, **5**, **7**–**9**, and **11**–**13** showed the moderate activity on the inhibition of AChE with the IC_50_ values ranging from 54.4 to 94.6 μM, compared with berberine (IC_50o_ of 0.67 μM). Regarding to the BChE inhibition, compound **13** showed the inhibitory effects against BChE with an IC_50_ value of 42.7 μM, compared with the positive control berberine (IC_50_ of 24.5 μM).

**Table 2:** Cholinesterase inhibitory activity of the isolated compounds

Compounds	IC_50_ (μM)^a^		Compounds	IC_50_ (μM)^a^	
	AChE	BChE		AChE	BChE
**1**	79.5 ± 1.1	>100	**11**	86.5 ± 0.6	>100
**2**	75.7 ± 3.1	>100	**12**	77.3 ± 0.8	>100
**3**	>100	>100	**13**	54.4 ± 3.4	42.7 ± 7.6
**4**	93.4 ± 2.2	–	**14**	>100	>100
**5**	83.9 ± 0.6	>100	**15**	>100	–
**6**	–	–	**16**	>100	>100
**7**	62.0 ± 2.2	–	**17**	>100	–
**8**	94.6 ± 1.5	>100	**18**	>100	>100
**9**	84.9 ± 1.2	>100	**19**	>100	–
**10**	>100	–	**20**	>100	–
**Berberine**	0.67 ± 0.0	24.5 ± 0.2	**21**	>100	–

^a^Data are the average of 3 replicates ± SD

**Fig. 3:**
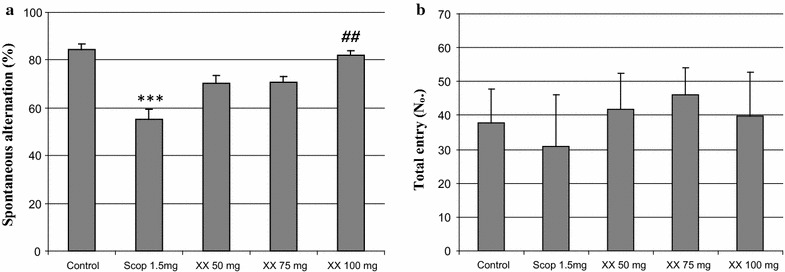
The effects of MEXX on scopolamine-induced memory impairment in mice in the Y-maze task. Spontaneous alternation behavior (**a**) and numbers of arm entries (**b**) during a 10 min session were recorded. Data represent mean ± SEM (n = 12 per group) (***P < 0.001 versus the vehicle-treated controls, ^##^ *P* < 0.01 versus the scopolamine-treated group)

Since MEXX showed potent inhibition activity against ChE enzymes in the primary experiments with the IC_50_ value of 16.17 μg/mL, the in vivo effects of MEXX on scopolamine-induced memory deficit were investigated by using the Y-maze task. A significant group effect was observed in spontaneous alternation behaviors [F (4, 55) = 10.859, *P* < 0.001]. Spontaneous alternation (%) in the scopolamine-treated group was significantly lower than that in the vehicle-treated control group (Fig. 3a, *P* < 0.001), and this spontaneous alternation reduction was significantly ameliorated following MEXX administration (100 mg/kg, p.o.) (Fig. 3a, *P* < 0.01). However, the mean numbers of the arm entries were similar in all experimental groups (Fig. 3b), which demonstrated that locomotor activity was not affected by MEXX.

**Fig. 4:**
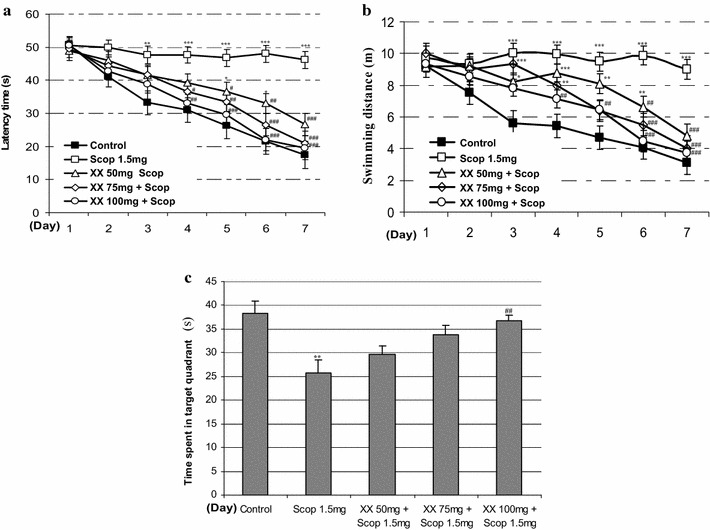
The effects of MEXX on escape latencies (**a**), and swimming distance (**b**) during the training-trial sessions and on swimming times during the probe-trial session (**c**) in the Morris water maze task on scopolamine induced memory dysfunction in mice. Data represent mean ± SEM (n = 12 per group) (*P < 0.05, **P < 0.01, ***P < 0.001 versus the vehicle-treated controls, ^##^ *P* < 0.01, ^###^ *P* < 0.001 versus the scopolamine-treated group)

Next, the effect of MEXX (50, 75 or 100 mg/kg, p.o.) on spatial learning was evaluated using the Morris water maze task. A repeated measures two-way ANOVA revealed that there were significant group effects for days [F (4.099, 45.088) = 46.944, *P* < 0.001], [F (3.788, 41.666) = 31.557, *P* < 0.001] and treatment groups [F (2.408, 26.483) = 34.871, *P* < 0.001], [F (3.555, 39.106) = 45.942, *P* < 0.001] on training-trial escape latencies and swimming distances, respectively. As shown in Fig. 2, the scopolamine-treated group (1.5 mg/kg, i.p.) exhibited longer escape latencies and swimming distances than did vehicle-treated controls from days 3 to 7 (Fig. 4a, b; *P* < 0.01 and *P* < 0.001). MEXX (50 mg/kg, p.o.) reduced escape latencies on day 5 (*P* < 0.05), day 6 (*P* < 0.01), day 7 (*P* < 0.001) and swimming distances on day 6 (*P* < 0.01), day 7 (*P* < 0.001) when compare to scopolamine-treated group. In addition, MEXX (75 mg/kg, p.o.) reduced escape latencies on day 4 (*P* < 0.05), day 5 (*P* < 0.01), day 6, 7 (*P* < 0.001) and swimming distances on day 5 (*P* < 0.01) day 6, 7 (*P* < 0.001) when compare to scopolamine-treated group. Finally, MEXX (100 mg/kg, p.o.) reduced escape latencies on day 4 (*P* < 0.01), day 5, 6, 7 (*P* < 0.001) and swimming distances on day 4, 5 (*P* < 0.01) day 6, 7 (*P* < 0.001) when compare to scopolamine-treated group. On the last day (day 8), the time in the target quadrant in scopolamine treated mice was significantly reduced compared to that of the vehicle-treated controls (Fig. 4c, *P* < 0.05). Furthermore, the shorter time in the target quadrant induced by scopolamine was significantly reduced by MEXX (100 mg/kg, p.o.) (Fig. 4c, *P* < 0.05).

**Fig. 5:**
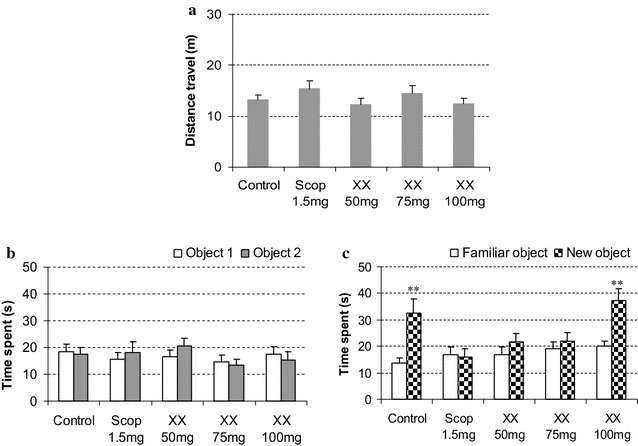
Effects of MEXX on object recognition deficits in mice in the sample phase (**b**) and the test phase (**c**), while data of locomotor activities are shown in (**a**). Each datum represents mean ± SEM (n = 12). The **P < 0.01 versus time spent exploring a familiar object (paired *t* test)

As shown in Fig. 5a, there was no significant difference in locomotor activities determined as total distance travel between vehicle-treated control, Scop 1.5 mg, and XX mice groups. Administrations of MEXX (50, 75 or 100 mg/kg, p.o.) before the experiments had no effect on locomotor activity compared with those in the vehicle-treated control. In the sample experiment, no mouse groups showed significant differences in time spent exploring each identical object (Fig. 5b). On the other hand, the control and XX 100 mg groups spent a significantly longer time exploring the new object than exploring the familiar one (*P* < 0.01 paired *t* test), while the XX 50 mg and XX 75 mg groups mouse showed a deficit in terms of the novel object recognition performance in the test phase session, as shown in Fig. 5c.

In this study, scopolamine significantly reduced spontaneous alternation (%) in Y-maze test and time exploring the new object in object recognition test in scop 1.5 mg group mice. These indicated that scopolamine induces impairment of short-term spatial and non-spatial working memory. In Morris water maze test, scopolamine impaired gradual decrease of escape latencies, swimming distances during training session and reduced the time spent in target quadrant during probe session. These observations suggest that scopolamine not only impairs the process of acquisition by producing anterograde amnesia, which subsequently affects the retrieval of these. Morris water maze test represents the model of memory especially spatial memory. During the training trials, mouse locates the hidden platform using spatial cues. This model is very helpful to analyze the reversal amnesic effect with investigational drug because receptive trials with ongoing trials confirm the progress of reversal of amnesia [27–29].

In our experiment, administration of MEXX plus scopolamine-treated groups showed significantly shorter mean escape latencies and swimming distances than did the scopolamine-treated group in training session. The swimming time of the scopolamine-treated mice within the platform quadrant was significantly reduced by treating with MEXX (100 mg/kg) in probe session. This indicated that MEXX is able to protect mice from scopolamine-induced learning and memory (both acquisition and retrieval process) impairment as assessed by the Morris water maze test. The in vitro inhibitory activity on AChE and BChE of MEXX suggesting that the in vivo memory enhancing effect of MEXX due to its AChE inhibition in cells and tissues. The results are in correlations with those of previous studies on the effect of memory enhancing of some natural product such as: Black Maca, imperatorin, *Lycium barbarum* polysaccharides [27, 30–32].

Working memory is one of the short-term memories that could be impaired at an early stage of AD [2, 29]. Previous reports have shown that Y-maze test is the experimental paradigms appropriate to evaluate anti-dementia activities of drugs including natural products [29, 33]. Some plants exhibit the inhibitory activity on AChE reduced spontaneous alternation (%) in Y-maze test [27, 34]. In our experiment, we employed Y-maze test to investigate effect of MEXX in short-term spatial working memory. The experimental results showed MEXX (100 mg/kg) improved scopolamine-induced decrease in spontaneous alternation (%) while it did not affect in spontaneous locomotors. This suggests that MEXX alleviated the memory impairment induced by scopolamine injection.

The effect of the MEXX on cognitive impairment was further confirmed by using object recognition test [35]. According to the results, no significant difference in total time spent exploring two identical objects was observed between control and scop 1.5 mg groups in sample phase session, indicating no differences in ability to recognize objects between animals. In the test phase session, the results showed that mice in the control group spent more time exploring the new object, whereas the scopolamine-treated mice showed no total time difference between familiar and new objects, indicating impairment of non-spatial object recognition memory. Administration of MEXX (100 mg/kg, p.o.) could significantly ameliorate scopolamin-induced recognition impairment against the new objects. This result is in correlation with other studies on *Ptychopetalum olacoides* [33], *Acanthopanax trifoliatus* [36], *Lycium barbarum* [31]. These plants inhibited AChE activity and improved performance in object recognition test in scopolamine treated mice.

Previous authors indicated that performance in Y-maze, object recognition task are impaired by anti-cholinergic drugs, as well as cholinergic neuronal lesions [32, 37, 38]. Conversely, improved performance in Y maze, object recognition was observed with drugs that enhance cholinergic activity, and inhibit AChE [27, 30]. Alzheimer’s treatment drug such as piracetam and pramiracetam, were shown to improve learning, memory and cognition in Morris water maze, Y-maze and object recognition test [38]. Our results are consistent with the notion that acetylcholine is critical in the processes underlying attention, learning and memory, the aging brain [3, 4].

## Methods

### General experimental procedures

The UV spectra were obtained with a Shimadzu UV-1800 recording spectrophotometer. The IR spectra were measured with a Shimadzu IR-408 spectrophotometer in CHCl_3_ solutions. The NMR spectra were taken on a Bruker Avance III 500 MHz spectrometer (Bruker Biospin) with tetramethylsilane (TMS) as an internal standard, and chemical shifts are expressed in *δ* values. The HR-ESI-MS was performed on a MicrO-QIITOF mass spectrometer (Bruker Daltonics). The ChE inhibitory reactions were recorded on 96-well microplates using a microplate reader (VersaMax ELISA, USA). Silica gel 60, 0.06–0.2 mm (70–230 mesh ASTM), for column chromatography was purchased from Scharlau (Barcelona, Spain). LiChroprep^®^ RP-18 (40–63 μm) for liquid chromatography was purchased from Merck KGaA (Germany). Analytical and preparative TLC were carried out on precoated Merck Kieselgel 60F_254_ or RP-18F_254_ plates (0.25 or 0.5 mm thickness).

### Animals and chemicals

Male Swiss mice (age, 8 weeks; weight, 25–27 g) were purchased from Military Medical University (Hanoi, Vietnam) and housed in a regulated environment (21 ± 2 °C, 12 h light/dark cycle, light period starting at 7 AM) with free access to food and water. Acetylcholinesterase (AChE) (EC 3.1.1.7), butyrylcholinesterase (BChE) (EC 3.1.1.8) and scopolamine hydrobromide (>98 %) were obtained from Sigma-Aldrich Pte Ltd (Nucleos, Singapore). Dithiobisnitrobenzoate (>99 %), berberine (>95 %) and DMSO were purchased from Merck (Darmstadt, Germany). Other chemicals were of the highest grade available.

### Plant material

The wood of *X. xylocarpa* was collected in Dak Lak province, Vietnam, in February 2012 and was identified by Dr. Truong LH, Southern Institute of Ecology, Vietnam Academy of Science and Technology. A voucher sample of the wood (P0046) has been deposited at the Department of Analytical Chemistry, Faculty of Chemistry, University of Science, Vietnam National University-Hochiminh City.

### Extraction and isolation

Dried wood (9.0 kg) of *X. xylocarpa* was extracted with MeOH (15 L, reflux, 3 h × 3) to yield 480 g of methanolic extract (MEXX). The MeOH extract was suspended in H_2_O and partitioned successively with hexane, EtOAc, and BuOH to yield hexane (21 g), EtOAc (53 g), BuOH (180 g), and remaining aqueous (226 g) fractions, respectively. The EtOAc fraction (53 g) was subjected to silica gel column chromatography (10 × 120 cm), eluted with MeOH/CHCl_3_ (0–50 %) yielding thirteen fractions (**fr.A**, 0.4 g; **fr.B**, 0.5 g; **fr.C**, 0.9 g; **fr.D**, 7.8 g; **fr.E**, 2.1 g; **fr.F**, 3.2 g; **fr.G**, 1.9 g; **fr.H**, 1.9 g; **fr.I**, 1.2 g; **fr.J**, 0.3 g; **fr.K**, 4.1 g; **fr.L**, 7.8 g and **fr.M**, 20.5 g). Fraction **fr.B** (0.5 g) was applied to silica gel column chromatography (2 × 80 cm), eluted with EtOAc/hexane (0–80 %) to give four subfractions (**fr.B1**–**B5**). Subfractions **fr.B2** and **fr.B3** were rechromatographed on a silica gel column with EtOAc/hexane as eluent to give compounds **2** (17.6 mg), and **12** (2.4 mg). Fraction **fr.C** (0.7 g) was also subjected to silica gel column chromatography (2 × 80 cm), eluted with EtOAc/hexane (0–80 %) to afford three subfractions (**fr.C1**–**C3**). Subfraction **fr.C1** was separated by column chromatography with EtOAc/hexane as eluent (0–60 %) and purified by preparative TLC to obtain **5** (3.5 mg) and **6** (6.3 mg). Subfraction **fr.C2** was further separated by silica gel column chromatography, eluted with EtOAc/hexane and CHCl_3_/hexane to give compound **11** (2.6 mg). Fraction **fr.D** (7.8 g) was dissolved in CHCl_3_/hexane (20:80) to gain the precipitation of **10** (2.4 g), the remaining part was further separated by silica gel column chromatography (5 × 80 cm) with EtOAc/hexane (0–80 %) to yield four subfractions (**fr.D1**–**D4**). Subfraction **fr.D1** was rechromatographed on silica gel column chromatography with EtOAc/hexane to give **3** (2.8 mg) and **8** (3.1 mg). Subfraction **fr.D3** was subjected to silica gel column chromatography and successively eluted with acetone/hexane (0–80 %), EtOAc/CHCl_3_ (0–50 %), acetone/CHCl_3_ (0–80 %), and then followed by preparative TLC with acetone/hexane (8:92) to afford **1** (15.7 mg), **4** (19.3 mg) and **9** (3.2 mg). Fraction **fr.E** (2.1 g) was separated by silica gel column chromatography (3 × 80 cm) with MeOH/CHCl_3_ (0–30 %) as eluent to yield four subfractions (**fr.E1**–**E4**). Subfractions **fr.E1** and **fr.E2** were purified by preparative TLC with EtOAc/hexane (20:80) and acetone/hexane (6:94) to yield **13** (14.6 mg), **14** (5.8 mg) and **15** (10.2 mg). Subfraction **fr.E3** was further separated by silica gel column chromatography with MeOH/CHCl_3_ to give four subfraction (**fr.E3.1**–**E3.4**). Subfraction **fr.E3.1** was rechromatographed on a silica gel column with EtOAc/hexane as eluent (0–60 %) to afford compound **16** (5.6 mg). The insoluble subfraction **fr.E3.4** was dissolved in acetone/hexane (10:90) and recrystallized to yield **18** (15.9 mg). Fraction **fr.F** (3.2 g) was further separated by silica gel column chromatography (3 × 80 cm) eluted with EtOAc/hexane (0–50 %) and MeOH/CHCl_3_ (0–30 %) and to give **19** (3.6 mg). Fraction **fr.G** (1.9 g) was subjected to silica gel column chromatography (3 × 80 cm) eluted with MeOH/CHCl_3_ (0–60 %) to give four subfractions (**fr.G1**–**G4**). Subfraction **fr.G1** and **fr.G2** was rechromatographed on silica gel column with EtOAc/hexane and CHCl_3_/hexane and respectively purified by preparative TLC with acetone/CHCl_3_ (10:90) and MeOH/CHCl_3_ (10:90) to give **7** (156.3 mg) and **21** (3.5 mg), respectively. Compound **19** (160 mg) was recrystallised from the insoluble fraction of **fr.I** (1.2 g) in acetone/hexane (10:90), and the remaining part was applied to silica gel column chromatography (2 × 80 cm) with MeOH/CHCl_3_ as eluent (0–50 %), the eluate was concentrated and crystallised in acetone/hexane (10:90) to afford **17** (10.2 mg).

*(3β)*-*Hopan*-*3*-*ol*-*28,22*-*olide (****1****)*: White amophous powder, IR (CHCl_3_) cm^−1^: 3310, 1730, 1170, 1100. ^1^H-NMR (CDCl_3_, 500 MHz) and ^13^C-NMR (CDCl_3_, 125 MHz), see Table 1. HR-ESI-MS *m/z*: 457.3674 [M + H]^+^ and 479.3482 [M + Na]^+^ (Calcd for C_30_H_49_O_3_, 457.3682; C_30_H_48_O_3_Na, 479.3501) (for further information, see Additional file 1).

### AChE and BChE inhibition assay

The inhibitory activities of the ChEs were measured using a modified Ellman’s method [39]. Acetylthiocholine and butyrylthiocholine were used as substrates to examine the inhibitory effect of sample on the AChE and BChE action, respectively. The reaction mixture contained: 140 μL of sodium phosphate buffer (pH 8.0); 20 μL of tested sample solution; and 20 µL of either AChE or BChE solution (5 units/mL), which were mixed and incubated at room temperature for 15 min. The reactions were initiated by the addition of 10 µL of dithiobisnitrobenzoate (DTNB) and 10 μL of either acetylthiocholine or butyrylthiocholine, respectively. The hydrolysis of AChE or BChE was monitored at 412 nm based on the formation of yellow 5-thio-2-nitrobenzoate anion from the reaction of DTNB with thiocholine, which was released by enzymatic hydrolysis of either AChE or BChE. All tested samples and the positive control, berberine [40], were dissolved in 10 % DMSO (analytical grade). The reaction was performed in triplicate and recorded in 96-well microplates using a microplate reader (VersaMax ELISA, USA). Percent inhibition was calculated from (1–*S*/*E*) × 100, where *E* and *S* were the enzyme activities with and without the tested sample, respectively. The ChE inhibitory activity of each sample was expressed in terms of the IC_50_ value (μM required to inhibit the hydrolysis of the substrate, AChE or BChE, by 50 %), as calculated from the logarithmic dose-inhibition curve.

### Animal grouping and drug treatment

The male Swiss mice were randomly assigned to five treatment groups (n = 12 per group): (**1**) Control (Saline), (**2**) Scop 1.5 mg (scopolamine 1.5 mg/kg/day), (**3**) XX 50 mg (MEXX 50 mg/kg/day + scopolamine 1.5 mg/kg/day), (**4**) XX 75 mg (MEXX 75 mg/kg/day + scopolamine 1.5 mg/kg/day) and (**5**) XX 100 mg (MEXX 100 mg/kg/day + scopolamine 1.5 mg/kg/day). MEXX was dissolved in saline and administered by oral gavage (p.o.). Scopolamine was also dissolved in saline and administered by intraperitoneal (i.p.) injection. MEXX was administered 60 min before the trial, and scopolamine was injected 30 min before the trial.

### Morris water Y-maze test

The Morris water maze is a black circular pool (80 cm in diameter and 35 cm in height) with a featureless inner surface. The circular pool was filled with water and nontoxic water-soluble black dye (20 ± 1 °C). The pool was divided into four quadrants of equal area. A transparent platform (4 cm in diameter and 18 cm in height) was centered in one of the four quadrants of the pool and submerged 1 cm below the water surface so that it was invisible at water level. The pool was located in a test room, which contained various prominent visual cues. The position of platform for escape and the visual cues remained unchanged throughout the experiments. The location of each swimming mouse, from the start position to the platform, was monitored by a video tracking system (ANY-maze, Stoelting, USA). In the water maze experiments, the day prior to the experiment was dedicated to swim training for 60 s in the absence of the platform. During the seven subsequent days, the mice were given four training-trials per session per day and an inter-trial interval of 2 min. For each training-trial, mice were placed in the water facing the pool wall in a randomly selected pool quadrant, the escape latencies and distance swim were recorded. These parameters were averaged for each day and for each mouse. Once the mouse located the platform, it was permitted to remain on it for 10 s. If the mouse did not locate the platform within 60 s, it was placed on the platform for 10 s and then removed from the. On day 8, the probe test involved removing the platform from the pool. That test was performed with the cut-off time of 120 s. The point of entry of the mouse into the pool was changed each trial thereafter. Mice were treated with saline or MEXX (50, 75 or 100 mg/kg, p.o.) given before the training trial. After 30 min, amnesia was induced in mice with scopolamine (1.5 mg/kg) given intraperitoneal injection. All mice were tested for spatial memory 30 min after scopolamine treatment.

### Y-maze test

The Y-maze is a three-arm maze with equal angles between all arms, which were 35 cm length and 5 cm width, with walls 10 cm high. The maze floor and walls were constructed from dark grey polyvinyl plastic. Mice were initially placed within one arm, and the sequence and number of arm entries were recorded 10-min period for each mouse and analyzed monitored by a video tracking system (ANY-maze, Stoelting, USA). One hour before the test, mice in control group and scop 1.5 mg group received distilled water and other mice were administered MEXX (50, 75, or 100 mg/kg, p.o.). After 30 min, memory impairment was induced by administering scopolamine (1.5 mg/kg, i.p.). Arms were cleaned between tests to remove odors and residues by diluted 10 % ethanol. Alternation behavior was determined from successive entries into three different arms (e.g., ABC, CAB, or BCA). An arm entry by the mice was defined as placing all four paws within a boundary of the arm. The alternation score (%) for each mouse was defined as the ratio of the actual number of alternations to the possible number (defined as the total number of arm entries minus two) multiplied by 100 as shown by the following equation: % Alternation = [(Number of alternations)/(Total arm entries − 2)] × 100. The number of arm entries was used as an indicator of locomotor activity.

### Object recognition test

The task took place in a to an open-field box (45 × 45 × 50 cm). Firstly, all animals were submitted to a habituation session, freely exploring the object free open field for 5 min. Twenty-four hours later, the sample phase session took place by placing individual mice for 5 min at the field in which two identical objects (A1 and A2; identical toys) were placed in a symmetrical position about 10 cm away from the wall; exploration was defined as the time spent sniffing or touching the object with the nose and/or forepaws. Test phase session were performed 24 h after training, when mice were allowed to explore the open field for 5 min in the presence of one familiar (A) and one novel (B) object. One hour before test phase session, mice were administered MEXX (50, 75, or 100 mg/kg, p.o.). The control group received distilled water. After 30 min, memory impairment was induced by administering scopolamine (1.5 mg/kg, i.p.). All objects presented similar textures and sizes, but distinctive shapes; after each trial objects were washed with 10 % ethanol to discard smells or residues. The exploration time and frequencies were recorded, n = 12 per group.

### Statistical analysis

The results of the behavioral studies are expressed as mean ± SEM, Y-maze test spontaneous alternation (%), object recognition test distance travel and Morris water maze test probe-trial swimming times were analyzed by one-way analysis of variance (ANOVA) followed by Tukey’s post hoc for multiple comparisons. The object recognition test time spent exploring a familiar and novel object in sample and test phase were analyzed by pair t-test. The Morris water maze test training-trial escape latencies and distance were analyzed by two-way ANOVA repeated followed by Tukey’s post hoc analysis using the day as one variable and treatment as a second. Statistical significance was set at *P* < 0.05.

## Conclusions

In conclusion, a new hopan-type triterpenoid, (3*β*)-hopan-3-ol-28,22-olide (**1**) was isolated together with twenty known compounds (**2**–**21**). Compound **1**, **2**, **4**, **5**, **7**–**9**, and **11**–**13** exhibited potent acetylcholinesterase (AChE); and compound **13** was also shown anti-butyrylcholinesterase (BChE) activity. The cognitive-enhancing effect of the MEXX on amnesic mice induced by scopolamine in vivo. It is noteworthy that MEXX is effective when administered orally to mice, experimental results are consistent with the traditional use of this medicinal plant species, the data here reported justify further studies with this plant extract in the context of treating attention and cognitive deficits associated with neurodegenerative diseases.

## Additional file

10.1186/s13065-016-0197-5 One-dimensional (1D) and two-dimensional (2D) nuclear magnetic resonance (NMR) and mass spectrometry (MS) of a new compound (**1**).
