# Supplementary material for: Anti-cholinesterases and memory improving effects of Vietnamese Xylia xylocarpa
Source: Chem Cent J. 2016 Aug 3;10:48. doi: 10.1186/s13065-016-0197-5 (PMC4973094; doi:10.1186/s13065-016-0197-5)
Supplement: Supplementary file 1 — 10.1186/s13065-016-0197-5 One-dimensional (1D) and two-dimensional (2D) nuclear magnetic resonance (NMR) and mass spectrometry (MS) of a new compound (1). [file 13065_2016_197_MOESM1_ESM.docx]

**Supplementary Files for**

**Anti-cholinesterase and memory improving effects of Vietnamese *Xylia xylocarpa***

Linh My Thi Lam^1^, Mai Thanh Thi Nguyen^1,*^, Hai Xuan Nguyen^1^, Phu Hoang Dang^1^, Nhan Trung Nguyen^1^, Tran Manh Hung^1^, Hoa Thi Nguyen^2^, Nui Minh Nguyen^2^, Byung Sun Min^3^, Jeong Ah Kim^4^, Jae Sue Choi^5^, Mao Van Can^2,**^

*^1^ Faculty of Chemistry, University of Science, Vietnam National University - Hochiminh City, 227 Nguyen Van Cu, District 5, Hochiminh City, Vietnam*

*^2^ Vietnam Military Medical University, Hadong District, Hanoi, Vietnam*

*^3^ College of Pharmacy, Catholic University of Deagu, Kyeongbuk 712-702, Korea*

*^4^ College of Pharmacy, Research Institute of Pharmaceutical Sciences, Kyungpook National University, Daegu 702-701, Korea*

*^5^ Department of Food Science and Nutrition, Pukyong National University, Busan 608-737, Korea*

* Corresponding author:

Assoc. Prof. Nguyen Thi Thanh Mai

*Faculty of Chemistry*

*University of Science, Vietnam National University - Hochiminh City*

*227 Nguyen Van Cu, District 5, Hochiminh City, Vietnam*

[nttmai@hcmus.edu.vn](mailto:nttmai@hcmus.edu.vn).

** Corresponding author:

Dr. Mao Van Can

*Vietnam Military Medical University, Hadong District, Hanoi, Vietnam*

*160, Phung Hung Street, Phuc La Ward, Hadong District, Hanoi, VietNam*

[canvanmao@yahoo.com](mailto:canvanmao@yahoo.com).

**CONTENTS:**

**Figure S1.1.** ^1^H-NMR spectrum of the compound **1** (500 MHz – CDCl_3_)

**Figure S1.2.** ^13^C-NMR spectrum of the compound **1** (125 MHz – CDCl_3_)

**Figure S1.3.** COSY-NMR spectrum of the compound **1**

**Figure S1.4.** HSQC-NMR spectrum of the compound **1**

**Figure S1.5.** HMBC-NMR spectrum of the compound **1**

**Figure S1.6.** NOESY-NMR spectrum of the compound **1**

**Figure S1.7.** HR-ESI-MS of the compound **1**

**Figure S1.1.** ^1^H-NMR spectrum of the compound **1** (500 MHz – CDCl_3_)

**Figure S1.2.** ^13^C-NMR spectrum of the compound **1** (125 MHz – CDCl_3_)

**Figure S1.3.** COSY-NMR spectrum of the compound **1**

**Figure S1.4.** HSQC-NMR spectrum of the compound **1**

**Figure S1.5.** HMBC-NMR spectrum of the compound **1**

**Figure S1.6.** NOESY-NMR spectrum of the compound **1**

**Figure S1.7.** HR-ESI-MS of the compound **1**
